# The effects of IQPLUS Focus on cognitive function, mood and endocrine response before and following acute exercise

**DOI:** 10.1186/1550-2783-8-16

**Published:** 2011-10-21

**Authors:** Adam G Parker, Josh Gordon, Aaron Thornton, Allyn Byars, John Lubker, Michelle Bartlett, Mike Byrd, Jonathan Oliver, Sunday Simbo, Chris Rasmussen, Mike Greenwood, Richard B Kreider

**Affiliations:** 1 Department of Kinesiology, Angelo State University, San Angelo, TX, 76909, USA; 2 Department of Sport and Exercise Science, West Texas A&M University, Canyon, TX, 79016, USA; 3 Department of Health and Kinesiology, Texas A&M University, College Station, TX, 77843, USA; 4 Graduate School, The University of Notre Dame, Notre Dame, IN, 46556, USA

**Keywords:** supplement, exercise, endocrine response, cognitive function, mood

## Abstract

**Background:**

Phosphatidylserine (PS) is a phospholipid found in cell membranes of most animals and plants. PS has been shown to reduce stress and increase performance in runners, cyclists and golfers. The purpose of this study was to investigate the effects of a PS containing formulation on cognitive function, mood and endocrine response before and after intense resistance exercise.

**Methods:**

18 lower body, resistance trained, college aged males ingested 14 days of supplement (IQPLUS Focus, providing 400 mg of soy-derived PS) and a Placebo (PL), in a randomized, double-blind, placebo controlled, cross-over manner. Following 14 days of supplementation, participants performed an acute bout of lower body resistance training. Mood (Profile of Mood States, POMS) and cognitive function (Serial Subtraction Test, SST) were measured prior to, 5 minutes after, and 60 minutes after exercise. Venous blood samples were collected prior to, and 5, 15, 25, 40 and 60 minutes after exercise. Blood samples were analyzed for plasma cortisol and testosterone. Data were analyzed using repeated measures ANOVA.

**Results:**

PS supplementation significantly reduced the time needed for a correct calculation on the SST by 20% (reduced by 1.27 s per calculation; PL: 6.4 s, PS: 5.13 s; p = 0.001), and reduced the total amount of errors by 39% (PL: 1.28 + .69, PS: .78 + .27, p = 0.53), and increased the amount of correct calculations by 13% (PL: 22.1 + 2.24, PS: 24.9 + 1.52, p = 0.07) prior to or in response to exercise compared to PL. Following exercise, there was no difference in SST scores between PS and PL. There were no significant changes in regards to mood or endocrine response to exercise as a result of PS supplementation.

**Conclusion:**

PS supplementation significantly increased cognitive function prior to exercise. Improved cognitive function could benefit athletes and non-athletes alike. PS did not appear to affect mood or endocrine response prior to or following resistance exercise.

## Background

It has been well documented that nutrients found in common food sources serve important functions in the human body. Many of these nutrients, like the essential vitamins and minerals we need every day, are required for survival. Other nutrients have not been deemed essential, however supplementation has been shown to be beneficial. One such nutrient is phosphatidylserine (PS). PS is a phospholipid found in cell membranes of most animals and plants [1]. In humans, PS is located in the internal layer of cell membranes where it serves many functions including regulation of receptors, enzymes, ion channels, and signaling molecules [1]. It is via these functions that PS may alter endocrine and cognitive function. Westerners ingest PS regularly in their diets in the amount of approximately 130 mg/day [1]. However, higher intake levels of PS through supplementation has been shown to be more beneficial than what is normally ingested from diet alone, improving age-related cognitive decline [2]. PS supplements have historically been derived from bovine brain tissue where it is particularly high in concentration, but due to health concerns related to the transfer of bovine spongiform encephalopathy (BSE), PS supplements for human consumption are now produced from soy phospholipids. There have been several studies that suggest supplementation with anywhere from 200-800 mg of PS per day can result in improved mood, cognitive functioning, sport performance, endocrine response to stress, and decreased soreness following exercise [1,3]. Short-term (10 days) high-dose (600 mg per day) supplementation with PS has been shown to attenuate cortisol response to moderate exercise via activiation of the hypothalamo-pituitary-adrenal axis [4] and low-dose (200 mg per day) long-term (6 weeks) consumption of PS and carbohydrates resulted in a reduction of perceived stress and improved golf performance [5]. Additionally, supplementation of 200 mg per day has been shown to induce a state of relaxation before and after exposure to a stressful environment [6]. By supplementing with PS, individuals may potentially be able to obtain better results from any exercise they participate in while at the same time improve mood and mental functioning. The purpose of this study was to determine if supplementation with PS (providing 400 mg of soy-derived PS) and a Placebo (PL) for 14 days, would improve cognitive performance, mood and/or endocrine response prior to and/or following a stress inducing bout of lower body, resistance exercise.

## Methods

### Experimental Approach to the Problem

Eighteen, physically active, college-aged males (N = 18, 22.5 ± 2.2 years of age, 1.77 ± .06 m, 84.4 ± 13.6 kg) ingested two servings of PS (IQPLUS Foods LLC, Milwaukee, WI, a proprietary formulation containing PS enriched soybean derived phospholipids, containing 200 mg of PS per serving) and a matching placebo (rice flour) for 14 days each (28 days total) in a random, placebo-controlled, double blind, cross-over design, with no washout period between supplements. Participants were deemed physically active if they had participated in lower body resistance exercise at least once per week for the prior 3 months. Participants were excluded from this investigation if they had any medical conditions that required prescription medication or prevented them from completing the exercise sessions. Participants were also not allowed to participate if they had consumed any nutritional supplement (except for a multivitamin/mineral) within the previous 30 days. All participants were informed of the requirements of the study and signed an informed consent form in compliance with the Guidelines for Research on Human Subjects of West Texas A&M University. Participant demographics are presented in table 1.

**Table 1:** This table shows demographic and strength data of the study participants.

Participant Demographics and Strength Measures	
Age	22.5 ± 2.2
Height (m)	1.77 ± .06
Weight (kg)	84.4 ± 13.6
Squat 1RM (kg)	125.2 ± 33.9
Leg Press 1RM (kg)	254.9 ± 80.2
Leg Extension 1RM (kg)	112.0 ± 26.9

Values are expressed as mean ± standard deviation.

### Familiarization

Participants in this study were asked to arrive at the Human Performance Research Laboratory (HPRL) at West Texas A&M University having fasted overnight. Participants underwent a fasting venous blood draw collected from the antecubital fossa, to determine pre-supplementation plasma cortisol and testosterone levels. Additionally, participants were required to perform 1 repetition maximum (RM) lifts in the Smith machine squat (SQ), leg press (LP), and leg extension (LE) exercises after performing a standardized warm up of walking briskly on a treadmill for five minutes. 1RM testing followed the National Strength and Conditioning Associations guidelines. Participants also performed a Serial Subtraction Test and a Profile of Mood States questionnaire to familiarize themselves with these instruments.

### Supplementation Protocol

Following familiarization, participants were randomly assigned to consume PS or PL for 14 days each. Following 14 days of supplementation with their first assigned supplement, participants reported to the HPRL for their first testing session. Upon completion of the first testing session, participants were given a 14 day supply of either PS or PL, depending on which supplement they took for the previous 14 days. No washout period was followed after the first supplementation period. After completing the 14 day supplementation period with the other supplement, participants reported to the HPRL for their second and final testing session.

### Cognitive Function and Mood Measurement

In order to analyze cognitive function, a Serial Subtraction Test (SST) was used. This consisted of a two minute timed test in which the participants subtracted the number 7 from a random 4 digit number in order to measure how quickly and accurately they can compute a simple mathematical problem. The average time per correct calculation, number of correct calculations, and number of mistakes were recorded. If an incorrect calculation was made, subsequent calculations were deemed correct based on the new starting number [7]. Analysis of mood was performed by administering the Profile of Mood States (POMS) questionnaire. The POMS measurement is used to measure transient mood states and measures six factors including: tension, depression, anger, fatigue, vigor, and confusion. The POMS has been used extensively in the past to examine changes in mood states as a result exercise [8].

### Testing Sessions

On both the first and second testing sessions, participants reported to the HPRL in a fasted state. In an attempt to account for diurnal variations in plasma cortisol levels, all testing sessions for each individual subject were scheduled for the same time of day, although that time varied between participants. Participants' heart rate and blood pressure were recorded, a pre-exercise (PRE) venous blood sample was collected, and a pre-exercise SST and POMS were collected. Following preliminary procedures, participants performed a 5 minute, whole body warm-up by walking briskly on a treadmill. Participants then performed 5 sets of 10 repetitions at 70% of their pre-determined 1RM for SQ, LP, and LE. Participants were allowed a 90 second rest between sets and a 180 second rest between exercises. This exercise protocol was determined to result in increases in plasma cortisol of approximately 87% in pilot testing. After completing the acute exercise bout, participants performed an SST and POMS at 5 and 60 minutes post-exercise (5POST and 60POST), and had venous blood samples collected at 5, 15, 25, 40, and 60 minutes post-exercise (5POST, 15POST, 25POST, 40POST, and 60 POST).

### Blood Analysis

All blood samples were collected via repeated venous blood draws from the antecubital fossa. Blood samples were centrifuged at 3, 400 rpm for 15 minutes, with the serum stored at -20°C for later analyses, as indicated in the instruction manual provided with the Enzyme Immunoassay (EIA) kits. Serum samples were then assayed for total testosterone and cortisol (Diagnostic System Laboratories, Webster, TX) viaEIA using an ELx808 microplate reader (BioTek Intruments, Inc., Winooski, VT) in the Exercise and Sport Nutrition Laboratory at Texas A&M University. All serum samples and reagents were brought to room temperature prior to analysis. 50 μL of each standard, control, and participant sample were added to their respective wells in addition to all required reagents. After the necessary incubation period, the absorbance of the solution in the wells was measured at 450 nm. A standard curve for concentration of serum cortisol and testosterone was developed via the data reduction software included with the microplate reader. Subject samples were compared to the standard curve to determine concentrations of cortisol and testosterone present.

### Statistical Analyses

SST data were analyzed using a 2 × 3 (treatment × time) repeated measures (RM) analysis of variance (ANOVA). POMS data were analyzed using a 2 × 3 (treatment × time) RM multiple analysis of variance (MANOVA). Serum cortisol and total testosterone data were analyzed using separate 2 × 6 (treatment × time) repeated measures ANOVAs. Bonferonni post-hoc procedures were used to compare means for any significant main effects or interactions. Additionally, paired samples t-tests were performed to compare SST results collected at PRE. Mauchly's test of sphericity was performed on all dependent variables with the Huynh-Feldt correction factor being utilized for any dependent variable that did not meet the assumption of sphericity. All statistical analyses were performed using SPSS 15.0 software for Windows (SPSS, Inc., Chicago, IL) with a probability level of p < 0.05 throughout.

## Results

### Cognitive Function

**Figure 1:**
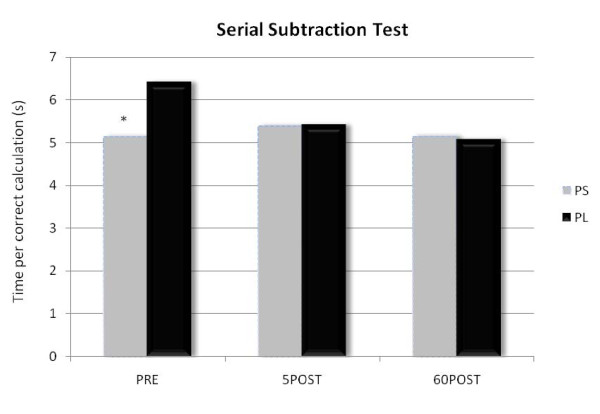
**Calculation speed**. Data are presented as seconds per correct calculation on the SST. * The PS group scored significantly lower at PRE compared to the PL group but not after exercise (p = 0.007).

SST data are presented in Figure 1. PS supplementation increased speed of calculation by 20% (PL: 6.44 ± 2.5 s, PS: 5.14 ± 1.3 s, p = 0.001), decreased the amount of mistakes made by 39% (PL: 1.28 ± .69, PS: .78 ± .27, p = 0.53), and increased the amount of correct calculations by 13% (PL: 22.1 ± 2.24, PS: 24.9 ± 1.52, p = 0.07) pre exercise. Statistical analysis revealed no significant treatment group differences (p > 0.05), however, there was a significant time × group interaction (p = 0.04), and a significant main effect for time (p = 0.045). Further statistical analysis revealed a significant reduction in time per correct calculation between supplement groups on the SST of approximately 20% (PL: 6.44 ± 2.5 s, PS: 5.14 ± 1.3 s, p = 0.007), and a significant decrease in time per correct calculation across both supplement groups between PRE and 60POST (5.1 ± 1.7 sec, p = 0.02).

### Mood

**Figure 2:**
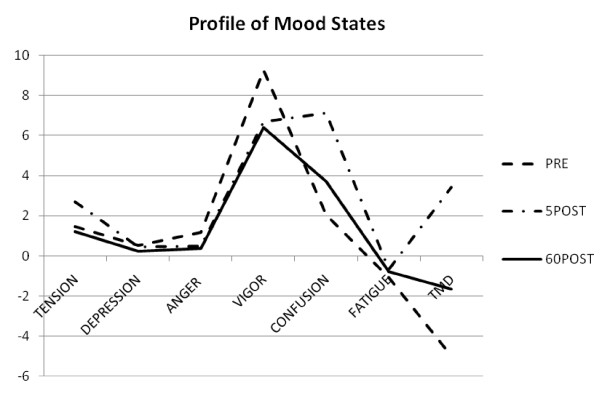
**This figure shows the mean POMS scores from both supplement groups combined to illustrate the effect exercise had on mood data**. There were no significant differences between supplement groups for POMS data (p > 0.05).

POMS data are presented in Figure 2. There were no significant differences between treatment groups or treatment × time interactions for any of the components of the POMS questionnaire (p > 0.05). The overall RM MANOVA for the POMS data resulted in a significant main effect for time. POMS results indicated a significant decrease in vigor from PRE to 5POST (p = 0.005) and 60POST (p = 0.000), as well as a significant increase in fatigue from PRE to 5POST (p = 0.014). Also, a significant increase for tension from PRE to 5POST (p = 0.049) with a significant decrease from PRE to 60POST (p = .000) and 5POST to 60POST (p = 0.031). Finally, total mood disturbance was significantly different between all three time points (p = 0.000).

### Endocrine Response

Endocrine data are presented in table 2. There were no significant differences between treatment groups or treatment × time interactions for serum cortisol, total testosterone, or the testosterone to cortisol ratio (p > 0.05). There was, however, a significant main effect for time for cortisol (p = 0.000) and testosterone (p = 0.004), indicating that exercise significantly increased both cortisol and testosterone levels across both treatment groups. Cortisol levels across time for both supplement groups were significantly higher at 5POST, 15POST, and 25POST compared to PRE (p = 0.001, p = 0.008, and p = 0.037 respectively), significantly lower at 40POST compared to 5POST, 15POST, and 25POST (p = 0.001, p = 0.001, and p = 0.003 respectively, and significantly lower at 60POST compared to 5POST, 15POST, 25POST, and 40POST (p = 0.000, p = 0.000, p = 0.000, and p = 0.008 respectively). Testosterone levels across time for both supplement groups were significantly higher at 5POST and 15POST compared to PRE (p = 0.034 and p = 0.002 respectively), and significantly lower at 60POST compared to 5POST and 15POST (p = 0.017 and p = 0.013 respectively).

**Table 2:** This table shows serum levels of total testosterone (ng/mL) and cortisol (μg/dL).

Endocrine Response						
	**PRE**	**5POST**	**15POST**	**25POST**	**40POST**	**60POST**

**CORTISOL**						
PL	17.2 ± 5.1	24.8 ± 9.4	25.4 ± 9.7	22.9 ± 9.5	19.7 ± 9.8	17.1 ± 8.2
PS	17.5 ± 7.1	28.8 ± 11.3	25.9 ± 11	24.2 ± 10.5	20.9 ± 10.7	19.5 ± 11.2

TESTOSTERONE						

PL	8.3 ± 2.9	11.3 ± 5.7	10.6 ± 3.5	9.7 ± 3.2	9.0 ± 2.1	8.6 ± 2.2
PS	8.9 ± 2.0	10.9 ± 3.6	10.8 ± 2.3	9.9 ± 1.7	9.9 ± 2.9	8.7 ± 3.2

Values are expressed as means ± standard deviation. There were no significant differences between supplement groups for serum total testosterone or cortisol (p > 0.05)

## Discussion

The results of this study have shown that supplementation with PS daily for 14 days significantly improved cognitive function prior to an acute bout of intense, lower-body resistance training. Supplementation with PS had no effect on mood, serum cortisol, or serum total testosterone. There were also no negative side-effects reported by any of the study participants in regards to PS supplementation.

Previous research has shown evidence that cognitive function may be improved by supplementing with PS. Baumeister et al., concluded that 42 days of supplementation with 200 mg of PS resulted in changes in electroencephalogram (EEG) activity indicating a more relaxed state following induced stress. This particular study also examined cognitive function using the Stroop colour-word interference test and the D2 concentration test. Despite the fact that the participants in this study had improved EEG readings, there was no evidence of significant differences in the measures of cognitive function as observed in our study [6]. According to a review article investigating the findings of PS supplementation in humans, Jäger et al., reported that significant improvements in cognitive function have been observed in elderly populations, but not in younger populations [1]. Additionally, an experiment by Jäger and colleagues found that golfers had improved golf performance following 42 days of supplementation with 200 mg of PS and 15 g of carbohydrates [5]. This improvement in performance may potentially be related to the relaxation effect observed in the study by Baumeister. It is possible that a relaxed mind may be able to better focus on sports tasks that require a great deal of concentration on sport skill performance, thus resulting in improved performance. According to our research, it seems that the most beneficial effect of PS supplementation is improvement in cognitive function prior to exercise that could potentially translate into improved performance in sports requiring a relaxed state of mind. Further research is warranted to examine the effects of PS supplementation on sport performance.

Our findings did not show any significant changes in mood states as measured by the POMS. An article by Benton et al. reported that young adults who scored high in measures of neuroticism experienced feeling less stress and had a better mood after PS supplementation of 300 mg/day for one month [9]. Another study investigated the effects of three different doses of PS (400, 600, or 800 mg/day for 21 days) on pituitary adrenal reactivity and the psychological response to a mental and emotional stressor [10]. It was observed that the 400 mg/day supplementation level resulted in an attenuated serum adrenocorticotropic hormone and cortisol, and salivary cortisol response to the stressor, as well as a decrease in distress. These effects were not seen in the other PS supplementation groups (600 or 800 mg/day). The results of our study showed that 14 days of supplementation with 400 mg of PS had no effect on serum cortisol or total testosterone levels. There have been numerous articles published reporting that PS supplementation can affect endocrine function, specifically by blunting cortisol response to stress [3,10,11]. However, several studies have also reported no changes in endocrine function as a result of PS supplementation [12,13]. Very few studies have been performed examining the effects of PS supplementation on testosterone levels. In one article, Starks found no significant changes in testosterone levels after 10 days of supplementation with 600 mg of PS [4]. These equivocal findings on mood and endocrine response have been attributed to differences in training status, dose and duration of supplementation and the kind of physical and mental stress [1,13]. Due to the strenuous nature of the exercise protocol used in this study, only resistance trained individuals were allowed to participate. The lack of significant changes to endocrine response between supplement groups may be due to the fact that the participants were not placed under an adequate amount of physical stress to elicit large enough changes in cortisol or testosterone levels. Perhaps more research is warranted to examine the effects of varying levels of both mental and physical stress on trained and untrained individuals to identify the populations that could benefit most from supplementation with PS.

## Conclusions

Supplementation with PS is an effective means of improving cognitive function in young, healthy college students. PS significantly increased the speed of calculations by 20%, reduced the total amount of errors by 39% and increased the total amount of correct calculations by 13%. Supplementation with PS did not have any significant effect on cortisol, total testosterone, or mood. This study is the first to suggest that a soy-derived PS formulation significantly increased mental performance in young adults prior to exercise, contributing to the growing body of evidence suggesting that soy-derived PS benefits cognition. Further research may be directed at determining the optimum dose of PS to achieve favorable endocrine response in athletes.

## Competing interests

The authors declare that they have no competing interests.

## Authors' contributions

AP was the primary author and carried out data collection, analysis of blood samples, and statistical analysis. JG and AT helped collect data. AB assisted with statistical analysis. JL and MB assisted with analysis of POMS and SST data. MG and RK assisted with manuscript preparation. MB, JO, SS, and CR assisted with analysis of blood samples. All authors read and approved the final manuscript.
